# First-Person Perspective Effects on Theory of Mind without Self-Reference

**DOI:** 10.1371/journal.pone.0019320

**Published:** 2011-04-29

**Authors:** Yuki Otsuka, Naoyuki Osaka, Ken Yaoi, Mariko Osaka

**Affiliations:** 1 Department of Psychology, Graduate School of Letters, Kyoto University, Kyoto, Japan; 2 Department of Psychology, Osaka University, Suita, Japan; Kyushu University, Japan

## Abstract

This study examined dissociations between brain networks involved in theory of mind, which is needed for guessing others' mental states, and the self, which might constitute the basis for theory of mind's development. We used event-related fMRI to compare a condition that required participants to guess the mental state of a subject featured in first-person perspective sentences (1stPP condition) with a third-person perspective sentence condition (3rdPP condition). The caudate nucleus was marginally more activated in the 1stPP than in the 3rdPP condition, while the left dorsolateral prefrontal cortex (DLPFC) was significantly more activated in the 3rdPP condition as compared to the 1stPP condition. Furthermore, we examined the correlation between activation (signal intensity) of the caudate nucleus and left DLPFC with that of the right DLPFC, which is thought to be closely connected with sense of self. We found a significant correlation between caudate nucleus and right DLPFC activation in the 1stPP condition, and between left and right DLPFC activation in the 3rdPP condition. Although theory of mind and the self both appear to recruit the right DLPFC, this region seems to be accessed through the left DLPFC during theory of mind tasks, but through the caudate nucleus when tasks require self reference.

## Introduction

When people communicate with one other, they are typically aware of the presence of the other person's mind, and are likely to attempt to guess at the nature of the other person's mental state. This process is thought to require theory –of mind (ToM). Several studies have recently investigated the neural basis of ToM [1], [2], [3], [4], [5], [6]. Frith and Frith [1] proposed that ToM seems to be mediated by the medial prefrontal cortex (MPFC), including the anterior cingulate cortex (ACC), temporal pole, and posterior superior temporal sulcus (STS). In addition to these brain areas, subsequent studies have indicated that the temporo-parietal junction (TPJ), inferior frontal gyrus (IFG) and anterior STS may also mediate ToM [3], [5], [7], [8].

The concept of the “self” is likely relevant to revealing the neural basis of ToM, given that ToM could not be acquired without prior development of the self [9]. This idea is supported by the findings that animals which display self-recognition (e.g., chimpanzees) pass many tasks that require ToM [10], [11], [12], [13], and that human infants seem to acquire ToM later than self-recognition [14], [15]. If ToM requires a developed sense of self as an initial basis, the neural basis of ToM is likely to be based (at least in part) on the neural basis of the self. Consistent with this view, some overlap in activation (mainly in the MPFC) is typically observed during ToM tasks on the one hand, and tasks that reference the self on the other [16], [17].

The precise extent to which the neural bases of ToM and the self overlap nevertheless remains up for debate. It was recently revealed that the so-called “E-network” mediates various functions, including sense of self, resting state, ToM, memory recall, and reasoning [16]. The E-network is an extensive cerebral network that includes the MPFC, precuneus, TPJ and temporal pole. The E-network may therefore serve as a common neural basis of ToM and the self. This proposal suggests the possibility that developmental processes that influence the E-network would also affect acquisition of both ToM and the self. In contrast, there is a view that the right PFC mediates sense of self, whereas ToM is mediated largely by the left PFC [18], [19], [20]. This view is supported by the observation that patients who sustained an injury to the right PFC show impairments in autobiographical memory, whereas patients with left PFC damage do not [21], [22]. However, neuroimaging studies have found right PFC activation during ToM tasks [23]. Keenan et al. [9] suggested the possibility that self is a fundamental prerequisite for the development of ToM. According to this view, the neural basis of ToM and the self would overlap at the right PFC, but the two bases would not show *complete* overlap.

As outlined above, there is no consensus regarding whether the neural basis of ToM would overlap completely with that of the self, although the issue is crucial for understanding the development of ToM. To examine possible differences in neural basis between ToM and the self, we used a task that required participants to guess at the mental state of an imaginary person featured in a short sentence written from the 1^st^ person perspective, without the requirement of self reference. The first-person-perspective (1stPP) pronoun “I” is usually used in connection with oneself (particularly in writing and talking). If a self-specific neural module exists in the brain, the 1stPP would be quite likely to tap into such a neural basis, and to do so more strongly than the third-person-perspective (3rdPP). The present study used fMRI to examine brain activity during a condition that required participants to guess about another's mental state based on sentences written from the 1stPP, with “I” as the subject (1stPP condition). This condition was compared to another that required participants to guess at another's mental state based on sentences written from the 3rdPP (using the pronouns “he” and “she”). We expected to observe different patterns of brain activity across the 1stPP and 3rdPP conditions, if indeed ToM and the self have separate neural bases. Furthermore, based on previous studies, we expected that the right PFC would show higher activation during the 1stPP condition as compared to the 3rdPP condition, whereas the left PFC should show the opposite pattern.

## Methods

### Participants

Twenty-two right-handed graduate and undergraduate students from Kyoto University, Osaka University of Foreign Studies, and Senri Kinran University (5 men and 17 women; mean age = 22.3 years, range = 19–29) participated in this study. All participants gave their written informed consent before the experiment, which was approved by the Ethics Committee of Advanced Telecommunications Research Institute International (ATR), Japan.

### Administered Tasks

The time course of each trial was fixed at 20 s, with each trial proceeding as follows. A start cue was initially presented for 500 ms and was followed by a sentence presented for 5200 ms, an inter-stimuli-interval of 500 ms, and a second sentence presented for 5200 ms. Immediately after the second sentence disappeared, a cue requiring a response from the participant appeared. The cue disappeared with the participant's response (YES or NO), and a blank screen appeared until the trial concluded.

We employed a task employed in previous studies [8], [24] that required participants to judge whether or not the second sentence presented was contextually consistent with the first sentence, based on a character's mental state. The subject of the first sentence was ‘I’ in the 1stPP condition, while “he” or “she” was used in the 3rdPP condition. Table 1 shows examples of the sentences used in the two conditions.

**Table 1:** Sentence examples for each condition.

1stPP		3rdPP	
I prepared three alarms on the bedside.	The National Center Test will begin at eight tomorrow.	He wiped sweaty palms on his trousers.	The final interview for new job will begin soon.
I wiped sweaty palms on my trousers.	The National Center Test will begin at eight tomorrow.	She prepared three alarms on the bedside.	The final interview for new job will begin soon.

Abbreviations: 1stPP = first-person-perspective; 3rdPP = third-person-perspective.

For our event-related design, we intermixed the stimuli from each condition as follows. We first created four lists of 78 sentence pairs, in which 18 different pairs were randomly assigned as 3rdPP stimuli. Within each list, half of the stimuli were original pairs and the remaining half constituted mixed pairs. Trial order was randomized separately for each condition. Condition order was pseudorandomized, with the constraint that not more than three consecutive trials would appear in the same condition. The experiment consisted of 78 trials in total.

### fMRI data acquisition

A 1.5-T fMRI scanner (Shimadzu-Marconi Magnex Eclipse) was used to acquire imaging data. Head movement was minimized using a forehead strap and soft pads positioned under the head. Twenty functional images with a thickness of 6 mm were acquired using the following parameters: TR, 2000 ms; TE, 48 ms; flip angle, 80°; FOV, 256×256 mm; and voxel size, 4×4×6 mm. Anatomical images were acquired after the experiment was completed, using the following parameters: TR, 12 ms; TE, 4.5 ms; flip angle, 20°; FOV, 256×256 mm; and voxel size, 1×1×1 mm. Stimuli were generated and synchronized using Presentation software (Neurobehavioral Systems, San Francisco, CA). Subjects viewed the stimuli on a projection screen via a mirror.

### fMRI data analysis

Data were analyzed using SPM5 (Wellcome Department of Cognitive Neurology, London, UK) in MATLAB (MathWorks, Sherborn, MA). The first six images in the scan sequence were excluded from analysis in order to rule out non-equilibrium effects of magnetization, and the remaining 841 functional image volumes were realigned to compensate for potential head movement related signal declination. As one participant showed head movements of >1 mm during the acquisition of functional images, images from the remaining twenty-one participants were analyzed. After realignment, the anatomical image was coregistered to the first volume of functional images. Functional images were then normalized with the anatomical image and spatially smoothed using a Gaussian filter (7 mm full-width half-maximum). Task-related activity was identified using the synthetic hemodynamic response function provided by SPM. For the event-related model, we time-locked the BOLD responses 9500 ms after the onset of the first sentence, based on the findings of a previous study [24]. Data were high-pass filtered with a frequency cut-off set at 32 s, the duration of the task alternation period, and low-pass filtered using a hemodynamic response function. A random effects model was applied, with a voxel-level threshold of *p*<0.001, uncorrected for multiple comparisons. We applied an uncorrected criterion because we focused on specific regions in which increased activation has previously been reported during similar tasks [24]. After non-linear transformation (http://imaging.mrc-cbu.cam.ac.uk/imaging/MniTalairach), we used the brain atlas of Talairach and Tournoux [25] to identify the activated anatomical regions.

Following identification of activated areas, percent signal changes in regions of interest (ROI) were obtained using MarsBaR [26]. We set ten brain areas as ROIs. These ROIs were selected based on a group average of the statistical maps. Each ROI and the center coordinate of spheres were set by intermediate local maxima between 1stPP and 3rdPP in common regions, or local maxima of each condition's unique region as follows: The bilateral dorsolateral prefrontal cortex (DLPFC; −43, 11, 40; 49, 21, 30), the bilateral IFG (−51, 17, 20; 50, 20, −8), the ACC (−8, 24, 38), the left anterior STS (−54, 8, −28), the left posterior STS (−62, −40, −4), the left superior parietal lobule (SPL; −33, −65, 38), the left TPJ (−55, −54, 19), the thalamus (−13, −12, 2), the globus pallidus (−22, −2, −4) and the caudate nucleus (CN; −12, 6, 10). ROIs were defined as spheres with radii of 3 mm, except for subcortical areas (thalamus, globus pallidus and the CN), which were defined as spheres with radii of 1 mm. We then used STATISTICA statistical software (version 06J, StatSoft, Tulsa, OK) to perform paired t-tests and correlational analyses of percent signal change for each ROI.

## Results

The behavioral data demonstrate high levels of response accuracy (1stPP, Mean = 90%, SD = 6.1, range = 75–98; 3rdPP, Mean = 86%, SD = 8.9, range = 67–100; *t*(20) = 2.38; *p*<0.05). Imaging data for all participants were therefore included in the following analysis.

**Figure 1:**
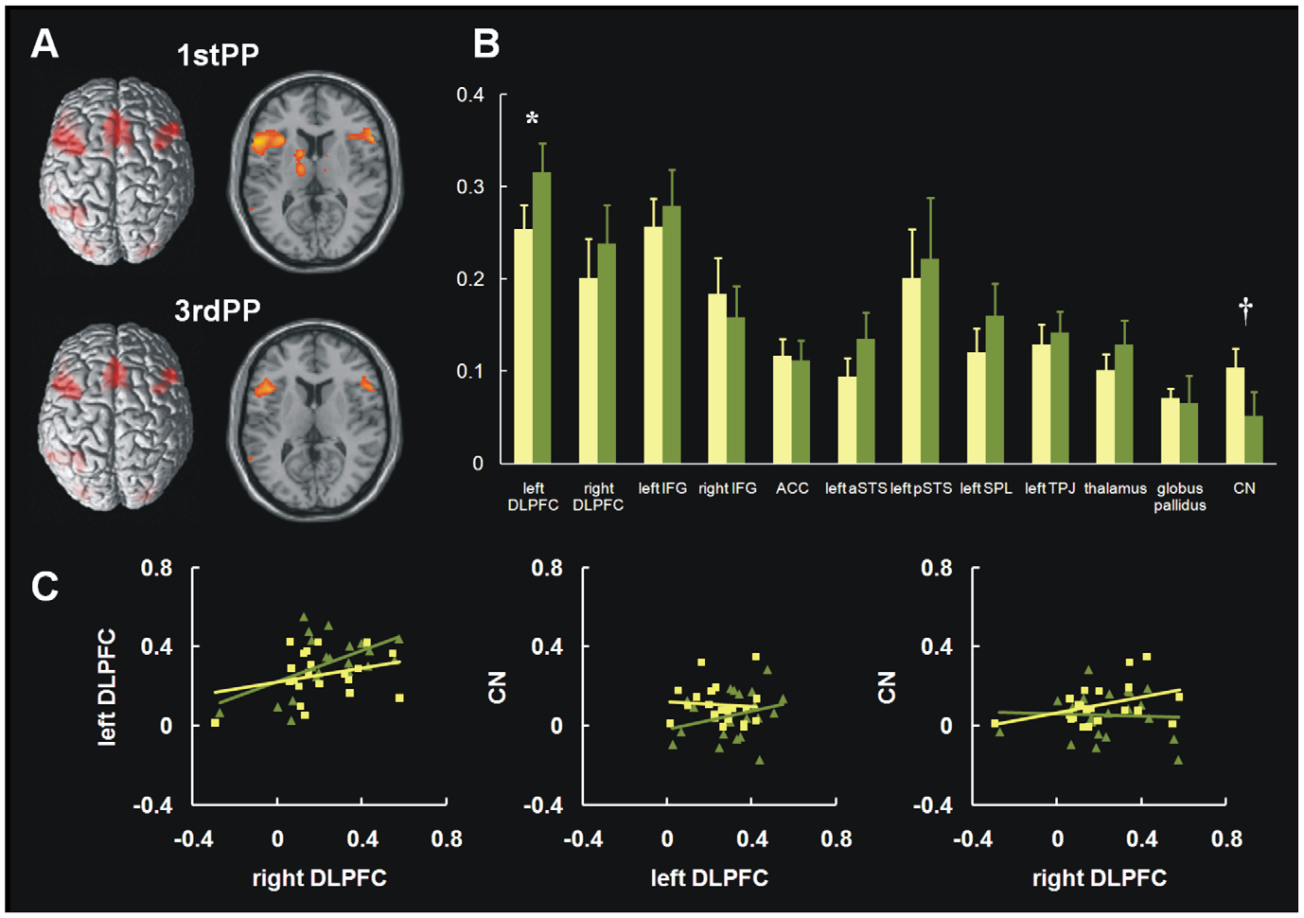
Activation areas for each condition superimposed on a 3D rendering of the brain and horizontal slice (right, z = 10). The threshold for significant activation was *p*<0.001 uncorrected at the cluster level (Panel A). Mean signal changes for each condition are shown for each region of interest (ROI). The yellow (left) bar indicates the first-person-perspective (1stPP) condition, and the green (right) bar indicates the third-person-perspective (3rdPP) condition. Abbreviations: 1stPP = first-person perspective; 3rdPP = third-person perspective; DLPFC = dorsolateral prefrontal cortex; IFG = inferior frontal gyrus; ACC = anterior cingulate cortex; aSTS = anterior superior temporal sulcus; pSTS = posterior superior temporal sulcus; SPL = superior parietal lobule; TPJ = temporo-parietal junction; CN = caudate nucleus. *, *p*<.05; †, *p*<.10 (Panel B). Percent signal change correlations between the left and right DLPFC (left panel), CN and left DLPFC (front panel), and right DLPFC and CN (right panel). Square points indicate the 1stPP condition and triangle points indicate the 3rdPP condition (Panel C).

Table 2 shows the main activation areas for each contrast. To examine signal intensity differences between the 1stPP and 3rdPP conditions, paired *t*-tests were conducted on mean signal changes for each ROI. Signal change in the left DLPFC was significantly higher for the 3rdPP condition (0.32±0.14%) than for the 1stPP condition [0.25±0.12; *t*(20) = 2.38, *p*<0.05]. In the CN, we found marginally higher signal change in the 1stPP condition (0.10±0.10%) as compared to the 3rdPP condition [0.05±0.12; *t*(20) = 2.07, *p* = 0.05]. Figure 1 displays the main activation areas and mean signal changes for each condition in each ROI (Panels A and B).

**Table 2:** Regions of activation during each condition.

		Coordinates				
Brain region activation	Brodmann's	x	y	z	T value	Voxels
1stPP						
middle frontal gyrus (DLPFC)	R9	44	22	32	4.86	1678
inferior frontal gyrus (VLPFC)	R45	56	22	18	6.15	
anterior cingulate cortex	L32	−8	26	38	6.7	1571
medial frontal gyrus	R6	8	14	54	5.87	
middle frontal gyrus (DLPFC)	L9	−44	12	40	9.82	4031
inferior frontal gyrus (VLPFC)	L45	−52	18	18	8.41	
posterior STS	L21	−60	−32	−8	5.08	188
superior parietal lobule	L7	−34	−66	38	5.03	990
temporoparietal junction	L22/39	−54	−54	20	6.16	
inferior parietal lobule	L7	−32	−56	42	5.97	
fusiform gyrus	L18	−24	−90	−14	9.8	950
inferior occipital gyrus	R18	26	−96	−6	6.67	404
thalamus		−12	−10	6	6.57	699
globus pallidus		−22	−2	−4	6.44	
caudate nucleus		−12	6	10	5.28	
3rdPP						
middle frontal gyrus (DLPFC)	R9	54	20	28	6.13	830
inferior frontal gyrus (VLPFC)	R45	54	24	−4	5.29	
anterior cingulate cortex	L32	−8	22	38	5.38	994
	L32	−10	10	42	6.14	
superior frontal gyrus	R8	4	26	48	8.29	
middle frontal gyrus (DLPFC)	L9	−42	10	40	10.6	2483
inferior frontal gyrus (VLPFC)	L47	−30	22	−8	7.48	
anterior STS	L21	−54	8	−28	3.78	
posterior STS	L21	−64	−48	0	4.69	829
temporoparietal junction	L22	−56	−54	18	6.33	
fusiform gyrus	R18	26	−94	−8	6.87	450
thalamus		−10	−10	4	5.56	172
1stPP-3rdPP						
middle frontal gyrus (DLPFC)	R46	30	46	28	4.96	39
middle frontal gyrus (DLPFC)	L9	−38	30	32	4.92	83
superior frontal gyrus	L10	−12	54	8	4.18	47
caudate nucleus		−18	20	2	5.33	39
3rdPP-1stPP						
no significant activation foci						

*Note*: uncorrected P<.001.

Abbreviations: 1stPP = first-person-perspective; 3rdPP = third-person-perspective; L = left; R = right; DLPFC = dorsolateral prefrontal cortex; VLPFC = ventrolateral prefrontal cortex; STS = superior temporal sulcus.

## Discussion

The present study used fMRI to compare brain activity during a condition that required participants to guess about another person's mental state on the basis of sentences written from the 1^st^ person perspective, with a similar task that involved sentences written from the 3^rd^ person perspective. If a self-specific neural basis (connected with the first-person-perspective, “I”) is in fact separate from the neural basis of ToM, different patterns of brain activity across the 1stPP and 3rdPP conditions would be expected, even without a clear self-reference requirement in the 1stPP condition. Based on previous findings, we also expected that the right PFC would show greater activation during the 1stPP condition as compared to the 3rdPP condition, whereas left PFC should show the opposite pattern. We found that signal intensity for the CN was marginally higher during the 1stPP condition than during the 3rdPP condition, whereas signal intensity for the left DLPFC showed the opposite pattern.

We found that use of the 1^st^ person perspective had ToM-related effects across our two conditions. This result seems to indicate the presence of a self-specific neural basis (connected with the first-person-perspective, “I”) that is separate from the neural basis of ToM. However, our results do not disconfirm the hypothesis that both ToM and sense of self recruit the E-network, which (as described earlier) consists of the MPFC (including ACC), precuneus, TPJ and temporal pole [16]. The ACC and TPJ showed no significant differences in signal intensity between the 1stPP and 3rdPP conditions, although we found significant activation of these regions during each condition. The E-network is probably shared by both ToM and the self, with further neural bases specific to each occurring outside of the E-network. Possible involvement of subcortical structures should receive greater consideration with regards to sense of self, given that we found self-related activity in the CN of the basal ganglia. Although this is not conclusive evidence that the self serves as the basis for development of ToM, the effect of the pronoun “I” (which does not require self-reference when guessing at another's mental state) may provide a clue: This effect indicates that ToM does not perfectly modulate the experience of self.

One unexpected result was that the CN showed higher activation during the 1stPP condition than during the 3rdPP condition, instead of the right PFC as earlier work would suggest. Previous studies have reported CN activation during subjective decision-making involving self reference [27], and in self-serving bias [28]. Blackwood et al. [28] proposed reward and implicit learning as possible reasons for the CN activation observed. Self-serving bias is quite likely to cause some form of internal reward, in that positive events tend to be more internally attributed than negative ones. However, it is difficult to envision how our experiment might have caused such a reward effect, given that we changed only the subject of the sentence across the 1stPP and 3rdPP conditions, without fundamentally changing sentence meanings. It therefore seems reasonable to suppose that CN activation would result from access to sense of self, formed via implicit learning. This possibility is consistent with the observed correlation between CN signal intensity and that for the right DLPFC. It must of course be noted that our findings might also be affected by our decision to examine implicit effects of the first-person perspective. Further research on the relationship between the CN and sense of self should help to clarify the neural basis of the latter.

Higher activation of the left DLPFC during the 3rdPP condition supports the view that left PFC activation during ToM tasks does not result from use of a verbal strategy [8]. ToM tasks often allow for the possibility that participants might have verbalized information in their minds, even when non-verbal stimuli are used. Accordingly, the issue of whether use of language causes the overlap that is often found between language and ToM networks has been discussed [29]. However, in the present study the same verbal stimuli were used in both 1stPP and 3rdPP conditions, except for the subject of the first sentence. The left DLPFC seems to show ToM-specific activation, and not only because we employed verbal stimuli.

### Conclusion

We used fMRI to examine a dissociation of the brain networks that underlie ToM and the self. Our findings showed that left DLPFC activity is related to ToM, and that caudate nucleus activity is related to sense of self.
